# Longitudinal association between somatic symptoms and suicidal ideation in adults with major depressive disorder

**DOI:** 10.3389/fpsyt.2025.1634899

**Published:** 2025-08-18

**Authors:** Subinuer Yiming, Yuhua Liao, Yanzhi Li, Wenjing Zhou, Hao Zhao, Ruiying Chen, Qindan Zhang, Yifeng Liu, Huimin Zhang, Christine E. Dri, Roger S. McIntyre, Wanxin Wang, Lan Guo, Beifang Fan, Ciyong Lu

**Affiliations:** ^1^ Department of Medical Statistics and Epidemiology, School of Public Health, Sun Yat-sen University, Guangzhou, China; ^2^ Department of Psychiatry, Shenzhen Nanshan Center for Chronic Disease Control, Shenzhen, China; ^3^ School of Public Health, Zunyi Medical University, Zunyi, China; ^4^ Brain and Cognition Discovery Foundation, Toronto, ON, Canada; ^5^ Department of Psychiatry, University of Toronto, Toronto, ON, Canada; ^6^ Department of Pharmacology and Toxicology, University of Toronto, Toronto, ON, Canada

**Keywords:** somatic symptoms, suicidal ideation, major depressive disorder, depression cohort in China, suicide prevention

## Abstract

**Background:**

Major depressive disorder (MDD) is often accompanied by somatic symptoms, but their longitudinal relationship with suicidal ideation (SI) remains insufficiently characterized. This longitudinal study in MDD patients aimed to (1) examine the associations between somatic symptoms (including total, pain, autonomic, energy, and CNS symptoms) and SI, and (2) investigate potential non-linear relationships among somatic symptoms and their subtypes with SI.

**Methods:**

Data was collected from patients with MDD in the Depression Cohort in China. The 28-item Somatic Symptoms Inventory (SSI) was used to assess somatic symptoms. SI was measured using the Beck Scale for Suicide Ideation (BSSI). Assessments were conducted at baseline and at weeks 4, 8, 12, 24, 48, and 72. Generalized estimating equations were utilized for exploring the associations of somatic symptoms and their subtypes with SI. GEE across three distinct models: Model 1 (unadjusted); Model 2 adjusted for sociodemographic and lifestyle factors; and Model 3 additionally adjusted for clinical characteristics. All models accounted for baseline SI.

**Results:**

These studies consisted of 1274 individuals with MDD (mean [SD], 27.7 [6.8] years; 399 (31.3%) males). The adjusted odds ratios (ORs) for SI across quartiles of total somatic symptom scores were 1.0 (reference), 0.95 (95% CI: 0.85-1.07, *P* = 0.419), 1.20 (95% CI: 1.03-1.41, *P* = 0.022), and 1.71 (95% CI: 1.39-2.11, *P* < 0.001) for quartiles 1,2, 3, and 4, respectively. Pain, autonomic, energy, and CNS symptoms showed similar results. A non-linear association (*P* for nonlinear < 0.001) was observed between total somatic symptom scores and SI. When the total somatic symptom score is below 49, the risk of SI remains at a relatively low level. However, when these scores exceeded the mentioned values, the risk of SI increases rapidly.

**Conclusions:**

Our findings suggest that in patients with MDD, there is a significant association between somatic symptoms and their subtypes with SI. Notably, the risk of SI is significantly increased by somatic symptoms in a nonlinear manner. These findings highlight the necessity of addressing somatic symptoms in the management of depression and emphasize the importance of developing targeted interventions to mitigate suicide risk in this vulnerable population.

## 1 Introduction

Major Depressive Disorder (MDD) is a prevalent mood disorder characterized by high prevalence, recurrent episodes, low remission rates, and high suicide rates (1–3). It is the second most prevalent cause of disability globally, affecting over 3.32 million individuals (4). MDD adversely affects not only social functioning and quality of life, but also significantly increases the risk of suicidal behaviors, thereby imposing substantial burdens on affected individuals, their families, and communities (5, 6). Suicide is the most severe consequence of depression. Globally, over 720,000 people die by suicide annually (7). An estimated 90% of individuals who die by suicide suffer from one or more mood disorders, with MDD accounting for 59-87% of all reported suicides (8). Suicidal ideation (SI) often presents prior to an index suicide attempt or fatality (9). In China, among individuals with MDD, the prevalence of SI is 27.5% over the past month and 53.1% over their lifetime (10). The greater the severity and pervasive the SI, the higher the likelihood that it will lead to an attempt (11). Therefore, it is necessary to further explore the risk factors for SI among patients with MDD in order to better prevent suicidal behaviors.

Somatic symptoms are common among MDD patients and present with diverse manifestations. In Western countries, approximately 66%–93% of individuals with MDD experience somatic symptoms of varying severity (12, 13). Under the influence of collectivist values, Chinese people are more likely to report somatic symptoms (14, 15). In China, over 70% of patients exhibit moderate to severe somatic symptoms (16). Even though certain somatic symptoms are not covered by the Diagnostic Statistical Manual (DSM-5), they often co-occur with depression (17). These symptoms are associated with more severe and longer-lasting depression, increased disability, poorer clinical outcomes, elevated medical expenses, and worse quality of life (12, 18–21). These factors also serve as independent risk factors for SI in individuals with MDD (22).

MDD patients with SI exhibit more frequent and severe somatic symptoms compared to those without SI (23). A Turkish study has shown that individuals with SI have an average of 20.1 somatic complaints, while those without SI have an average of 10.6 somatic complaints (24). Previous research has demonstrated a significant association between somatic symptoms and a heightened risk of SI, with this risk seeming to exist independently of the presence of concurrent mental disorders (17, 18). However, there is a lack of longitudinal research evidence regarding individuals with MDD, as well as an exploration of whether there is a non-linear relationship between somatic symptoms and SI.

Somatic symptoms in patients with depression can be categorized into different subtypes, including pain symptoms, autonomic nervous system symptoms, energy- symptoms, and central nervous system (CNS) symptoms (20, 25). Most prior studies on the association between somatic symptom subtypes and SI have focused on single symptoms within specific somatic subtypes. However, patients often experience multiple somatic symptoms simultaneously rather than just one. Therefore, considering only single symptoms when examining the relationship between somatic symptoms and SI is insufficient. The cumulative impact and overall burden of co-occurring somatic symptoms must also be taken into account. While some studies have concentrated on the overall burden of pain symptoms, research on other subtypes remains limited.

Therefore, this longitudinal study of MDD patients in China, evaluating the burden of somatic symptoms and their subtypes (including pain, autonomic, energy, and CNS symptoms), aimed to: (1) investigate the associations between somatic symptoms and their subtypes with SI, and (2) evaluate non-linear relationships among somatic symptoms and their subtypes with SI.

## 2 Methods

### 2.1 Study population

The data were sourced from the Depression Cohort in China (DCC; ChiCTR registry number: 1900022145) (26). The DCC is a large-scale, ongoing research project that focuses on individuals diagnosed with MDD. Participants were recruited from two mental health institutions in Shenzhen between June 2020 and September 2024. The diagnosis of MDD was confirmed through a structured clinical interview using the Mini-International Neuropsychiatric Interview (M.I.N.I.) conducted by psychiatrists. After enrollment, participants underwent a baseline assessment and were followed up at weeks 4, 8, 12, 24, 48, and 72. Data analysis included individuals who had at least one follow-up visit during the 72-week follow-up period.

Participants met the following inclusion criteria: (1) 18–65 years; (2) a score of ≥10 on the Patient Health Questionnaire-9 (PHQ-9) and ≥8 on the 17-item Hamilton Depression Rating Scale (HAMD-17); (3) demonstrated capacity for effective communication and provision of informed consent. Individuals were excluded if they met any of the following criteria: (1) diagnosed with severe mental comorbidities (e.g., bipolar disorder, schizophrenia); (2) combined with neurological diseases (e.g., epilepsy, encephalitis, traumatic brain injury); (3) have a history of substance abuse (such as alcohol, drugs); (4) Current pregnancy or lactation status. The Institutional Review Board of Sun Yat-sen University School of Public Health granted ethical approval for this protocol (Ethical code: L2017044). All participants provided written informed consent before initiating study procedures.

**Figure 1:**
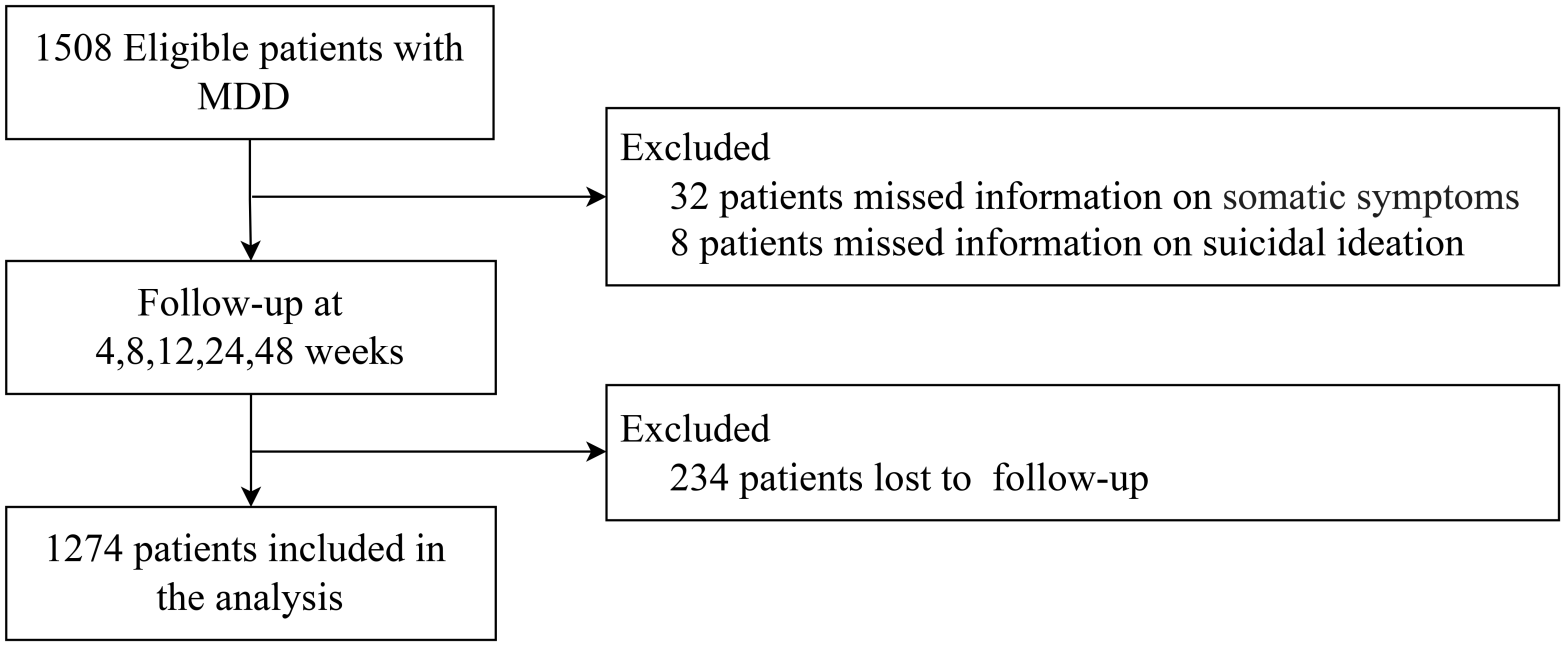
The inclusion and exclusion process of patients with major depressive disorder. MDD, Major depressive disorder.

The study enrolled a total of 1,508 participants diagnosed with MDD. Following the exclusion of individuals with incomplete data on somatic symptoms (n = 32), SI (n = 8)and those who were lost to follow-up (n = 234), the final analysis comprised 1274 patients with MDD. The process of participant selection and exclusion is illustrated in **Figure 1**.

### 2.2 Assessment of somatic symptoms

Somatic symptoms were evaluated using the 28-item Somatic Symptoms Inventory (SSI) during baseline and follow-up visits. The SSI is a self-assessment instrument that measures the severity of somatic symptoms reported by the individual throughout the preceding week (27, 28). The SSI shown good reliability in our study (McDonald’s omega = 0.96). Each item on the SSI was scored from 1 to 5 (1 = “absent”; 2 = “a little bit”; 3 = “moderate”; 4 = “quite a bit”; 5 = “a great deal”). The total score of the SSI ranges from 28 to 140, with a higher scores signifying greater severity of somatic symptoms (29). Based on previous studies (20, 25), Somatic symptoms were categorized into 4 subtypes, including pain symptoms (scoring range: 7 to 35), autonomic symptoms (scoring range: 11 to 55), energy symptoms (scoring range: 6 to 30), and CNS symptoms (scoring range: 4 to 20). Participants were stratified into four groups (Quartile 1–Quartile 4) according to quartiles of total somatic symptom scores and each subtype score.

### 2.3 Assessment of SI

The Beck Scale for Suicide Ideation (BSSI) was used to measure SI in the past week both at baseline and during follow-up visits (30, 31). This self-report instrument includes 19 items that are scored on a 3-point Likert scale, with scores ranging from 1 to 3 (1 = “I have no wish to die”; 2 = “I have a weak wish to die”; 3 = “I have a moderate-to-strong wish to die”). SI severity is evaluated using the first five items (32). The SI assessment has a score range of 5 to 15, where higher scores indicate more severe SI (33). The BSSI shown good reliability in our study (McDonald’s omega = 0.96).

### 2.4 Assessment of potential covariates

During baseline and follow-up evaluations, structured self-completed questionnaires were used to collect covariate data. These including sociodemographic factors, health-related factors and MDD-associated clinical characteristics.

sociodemographic factors included age, sex (male or female), ethnicity (Han or Other), education (high school or below; undergraduate; master’s degree or above), marital status (married; unmarried/divorced/widowed), employment status (unemployed or employed), monthly household income (no fixed income;<10–000 yuan; 10 000–19–999 yuan; ≥20–000 yuan) and living arrangements (living alone; living with families; living with others) (34, 35).

Health-related factors included lifetime smoking status (yes or no), lifetime drinking status (yes or no), weekly exercise habits (yes or no), comorbidity (yes or no)

, and body-mass index (BMI) (36). Lifetime smoking, lifetime drinking, and weekly exercise habits were evaluated by the following questions: “Have you ever smoked a cigarette (yes or no)?” “Have you ever consumed at least one alcoholic drink of any kind (yes or no)?” “Do you have a weekly exercise habit (yes or no)?”. Comorbidity (diagnosed at the community health service center or a higher level of medical institution, including all diseases suffered to date), (1=has a history of illness; 0=has no history of illness).

MDD-associated clinical characteristics included the severity of depressive symptoms, first episode (yes or no), severity of anxiety symptoms, current antidepressant use (yes or no), previous antidepressant use (yes or no), and sleep medication use (yes or no)

(20). The severity of depression symptoms during the past two weeks was assessed using the Patient Health Questionnaire-9 (PHQ-9). The severity of anxiety symptoms during the past two weeks was assessed using the Generalized Anxiety Disorder-7 (GAD-7). The assessment of first-episode MDD was conducted using the question: “How many depressive episodes have you experienced in total so far?” Participants who reporting only one depressive episode were classified as having a first-episode MDD.

### 2.5 Statistical analyses

Initially, we conducted descriptive analyses by grouping participants by sex to compare baseline characteristics between males and females. Continuous variables were described using mean (SD), and categorical variables were described using frequencies with percentages. Differences between the gender groups were compared using independent samples t-tests and chi-square tests.

Secondly, we applied Generalized Estimating Equations exploring the longitudinal relationship between somatic symptoms and its subtypes with SI across three models: Model 1 was unadjusted; Model 2 was adjusted for covariates, including sociodemographic factors (e.g., age, sex, ethnicity, education, marital status, employment status, monthly household income, and living arrangements) and health-related factors (e.g., lifetime smoking status, lifetime drinking status, weekly exercise habits, comorbidity, and BMI); and Model 3 further adjusted for MDD-associated clinical characteristics, such as the severity of depression symptoms, the severity of anxiety symptoms, first episode, current antidepressant use, previous antidepressant use, sleep medication use, and time trends. All models accounted for baseline SI. In the above analysis, an unstructured correlation working structure and a linear link function for the continuous outcome variable were employed. The unstructured working correlation matrix was selected because it yielded the lowest quasi-likelihood under the independence model criterion (QIC), indicating the best-fitting structure, and a linear link function was employed for the continuous outcome. We used multiple imputations with chained equations with 5 data sets to impute confounders with missing values, thereby reducing potential bias from missing covariate data. Additionally, Scores for total somatic symptoms and its subtypes were categorized into four groups according to their quartiles and subjected to a trend test.

Lastly, we used restricted cubic splines to further investigate the non-linear association of somatic symptoms and its subtypes with SI. Specifically, we applied restricted cubic splines with 4 knots positioned at the 5th, 35th, 65th, and 95th percentiles of the total somatic symptom score and the scores of its subtypes to model this relationship.

Statistical analyses were completed utilizing R software (version 4.1.3; R Foundation for Statistical Computing, Vienna, Austria). A two-sided P value below 0.05 was deemed to indicate statistical significance.

## 3 Results

### 3.1 Characteristics of study population

**Table 1** displays the characteristics of the study participants. A total of 1274 patients were included in the study, comprising 399 males (31.3%) and 875 females (94.87%), with a mean age of 27.7 years (SD, 6.77 years). Most participants were Han ethnic (94.87%), highly educated (83.39%), non-married (73.90%) and employed (67.74%). Participants whose monthly household income was ≥20,000 yuan accounted for the largest part (34.2%). Regarding health-related factors, the majority of the participants reported no exercise habits (64.23%), alcohol consumption (85.62%), no smoking cigarettes (57.27%), and lived with families (55.07%). Regarding clinical characteristics, more than half of the participants were at their first episodes of MDD (63.60%), were prescribed antidepressants (80.98%), were not prescribed sleep medications (73.03%), and did not have comorbidities (66.85%) at baseline. Educational levels, employment status, lifetime drinking status, living alone, antidepressant use, previous antidepressant use, first episode and comorbidity were balanced among participants within different groups. In addition, the mean (SD) scores for SI, total somatic symptom, pain symptom, CNS symptom, autonomic symptoms, energy symptoms, PHQ-9 and GAD-7 were 8.96 (2.6), 62.2 (21.3), 14.7 (6.04), 7.78 (3.37), 22.7 (8.54), 17.4 (6.07), 19.5 (4.49) and 14.1 (4.83) respectively.

**Table 1:** Baseline characteristics of the participants included.

Variable ^a^	Overall (1274)	Male (399)	Female (875)	*P*-value ^b^
Age, mean (SD), years	27.7 (6.77)	27.7 (6.74)	27.8 (6.79)	<0.001
Ethnicity (n,%)				0.043
Han	1202 (94.87)	384 (96.73)	818 (94.02)	
Other	65 (5.13)	13 (3.27)	52 (5.98)	
Education (n,%)				0.123
High school or below	211 (16.60)	78 (19.65)	133 (15.22)	
Undergraduate	920 (72.38)	274 (69.02)	646 (73.91)	
Master’s degree or above	140 (11.01)	45 (11.34)	95 (10.87)	
Marital status (n,%)				0.004
Married	332 (26.10)	83 (20.80)	249 (28.52)	
Unmarried/divorced/widowed	940 (73.90)	316 (79.20)	624 (71.48)	
Employment status (n,%)				0.770
Unemployed	410 (32.26)	130 (32.83)	280 (32.26)	
Employed	861 (67.74)	266 (67.17)	595 (68.00)	
Monthly household income (n,%)				0.048
No fixed income	145 (11.95)	59 (15.40)	86 (10.36)	
<10–000 yuan	318 (26.22)	88 (22.98)	230 (27.71)	
10 000–19–999 yuan	323 (26.63)	100 (26.11)	223 (26.87)	
≥20–000 yuan	427 (35.20)	136 (35.51)	291 (35.06)	
Living arrangements (n,%)				0.141
Living alone	329 (26.26)	116 (29.82)	213 (24.65)	
Living with families	690 (55.07)	201 (51.67)	489 (56.60)	
Living with others	234 (18.68)	72 (18.51)	162 (18.75)	
Lifetime smoking status (n,%)				<0.001
Yes	544 (42.73)	244 (61.31)	300 (34.29)	
No	729 (57.27)	154 (38.69)	575 (65.71)	
Lifetime drinking status (n,%)				0.112
Yes	1090 (85.62)	350 (87.94)	740 (84.57)	
No	183 (14.38)	48 (12.06)	135 (15.43)	
Weekly exercise habits (n,%)				<0.001
Yes	455 (35.77)	178 (44.61)	277 (21.78)	
No	817 (64.23)	221 (55.39)	596 (68.72)	
Comorbidity (n,%)				0.351
Yes	422 (33.15)	125 (31.33)	297 (33.98)	
No	851 (66.85)	274 (68.67)	577 (66.02)	
First episode (n,%)				0.913
Yes	809 (63.60)	254 (63.82)	555 (63.50)	
No	463 (36.40)	144 (36.18)	319 (36.50)	
Antidepressant use (n,%)				0.357
Yes	1005 (80.98)	325 (82.49)	680 (80.28)	
No	236 (19.02)	69 (17.51)	167 (19.72)	
Previous Antidepressant use (n,%)				0.787
Yes	445 (34.96)	137 (34.42)	308 (35.20)	
No	828 (65.04)	261 (65.58)	567 (64.80)	
Sleep medications use				0.913
Yes	342 (26.97)	100 (25.38)	242 (27.69)	
No	926 (73.03)	294 (74.62)	632 (72.31)	
BMI, mean (SD)	21.5 (3.83)	23.1 (4.03)	20.8 (3.51)	<0.001
PHQ-9 scores, mean (SD)	19.5 (4.49)	19.2 (4.48)	19.7 (4.49)	0.06
GAD-7 scores, mean (SD)	14.1 (4.83)	13.98 (4.90)	14.22 (4.81)	0.40
SI scores, mean (SD)	8.96 (2.6)	8.77 (2.59)	9.05 (2.60)	0.08
Total somatic symptom score, mean (SD)	62.2 (21.3)	58 (19.7)	64.7 (21.7)	<0.001
Pain symptoms score, mean (SD)	14.7 (6.04)	13.5 (5.59)	15.3 (6.15)	<0.001
CNS symptoms score, mean (SD)	7.78 (3.37)	7.5 (3.23)	7.9 (3.42)	0.04
Autonomic symptoms score, mean (SD)	22.7 (8.54)	20.7 (7.63)	23.6 (8.78)	<0.001
Energy symptoms score, mean (SD)	17.4 (6.07)	16.3 (5.86)	17.9 (6.10)	<0.001

a Unless otherwise indicated, data are expressed as No. (%) of participants.

b Baseline characteristics were compared between the two groups using two independent-sample *t*-tests for continuous variables and Chi-Square tests or Fisher exact probabilities for categorical variables.

BMI, body mass index; PHQ-9, Patient Health Questionnaire-9; GAD-7, General Anxiety Disorder-7; SI, suicidal ideation.

### 3.2 Relationship between somatic symptoms and SI

After adjusting for potential confounders, we discovered that somatic symptom quartiles were associated with SI, as shown in **Table 2**. compared with quartile 1 of total somatic symptom scores, the full-adjusted odds ratios (*ORs*) and 95% CIs for SI from quartile 2 to 4 were 1.0 (reference), 0.95 (95% CI: 0.85-1.07, P = 0.419), 1.20 (95% CI: 1.03-1.40, P = 0.022), and 1.71 (95% CI: 1.39-2.11, P < 0.001) for quartiles 1,2, 3, and 4, respectively. *P*-value for trend < 0.001. In the 4 and 3 quartiles of total somatic symptom scores, patients had 1.7 and 1.2 times the risk of SI compared to those in the 1 quartile. In contrast, there was no significant association between somatic symptoms and suicidal ideation in the second quartile. Across all somatic symptom subtypes consistent pattern was observed. Compared to quartile 1, the ORs for SI in quartiles 2–4 were 1.03 (95% CI: 0.92 - 1.14, P = 0.631), 1.22 (95% CI: 1.05 - 1.41, P = 0.010), and 1.67 (95% CI: 1.39 - 2.00, P < 0.001) for pain symptoms scores; 1.03 (95% CI: 0.93 - 1.14, P = 0.573), 1.24 (95% CI: 1.09 - 1.42, P = 0.002), and 1.80 (95% CI: 1.48 - 2.18, P < 0.001) for autonomic symptoms scores; 1.02 (95% CI: 0.91 - 1.14, P = 0.742), 1.17 (95% CI: 1.00 - 1.36, P = 0.048), and 1.74 (95% CI: 1.42 - 2.14, P < 0.001) for energy symptoms scores; and 1.03 (95% CI: 0.93 - 1.14, P = 0.616), 1.24 (95% CI: 1.09 - 1.41, P = 0.002), and 1.61 (95% CI: 1.33 - 1.96, P < 0.001) for CNS symptoms scores. All P - values for trend were below 0.001.

**Table 2:** Associations between somatic symptoms and their subcategories with SI.

Variable	Model 1		Model 2		Model 3	
	OR (95%CI)	*P*-value	OR (95%CI)	*P*-value	OR (95%CI)	*P*-value
Total somatic symptoms						
Quartile 1 (28–37)	0 [Reference]		0 [Reference]		0 [Reference]	
Quartile 2 (38–49)	1.76 (1.56-1.99)	<0.001	1.70(1.51-1.92)	<0.001	0.95 (0.85-1.07)	0.419
Quartile 3 (50–68)	3.64 (3.12-4.26)	<0.001	3.41 (2.92-3.98)	<0.001	1.20 (1.03-1.40)	0.022
Quartile 4 (69–140)	9.79 (7.94-12.07)	<0.001	9.09 (7.38-11.20)	<0.001	1.71 (1.39-2.11)	<0.001
P-value for trend		<0.001		<0.001		<0.001
Pain symptoms						
Quartile 1 (7–8)	0 [Reference]		0 [Reference]		0 [Reference]	
Quartile 2 (9–11)	1.56 (1.38-1.75)	<0.001	1.51 (1.35-1.7)	<0.001	1.03 (0.92-1.14)	0.631
Quartile 3 (12–16)	2.79 (2.38-3.28)	<0.001	2.63 (2.24-3.08)	<0.001	1.22 (1.05-1.41)	0.010
Quartile 4 (17–35)	6.5 (5.31-7.96)	<0.001	6.08 (4.98-7.42)	<0.001	1.67 (1.39-2.00)	<0.001
P-value for trend		<0.001		<0.001		<0.001
Autonomic symptoms						
Quartile 1 (11–13)	0 [Reference]		0 [Reference]		0 [Reference]	
Quartile 2 (14–17)	1.69 (1.51-1.89)	<0.001	1.65 (1.47-1.84)	<0.001	1.03 (0.93-1.14)	0.573
Quartile 3 (18–25)	3.1 (2.68-3.58)	<0.001	2.9 (2.51-3.35)	<0.001	1.24 (1.09-1.42)	0.002
Quartile 4 (26–55)	8.18 (6.61-10.12)	<0.001	7.59 (6.15-9.38)	<0.001	1.80 (1.48-2.18)	<0.001
P-value for trend		<0.001		<0.001		<0.001
Energy symptoms						
Quartile 1 (6–9)	0 [Reference]		0 [Reference]		0 [Reference]	
Quartile 2 (10–13)	1.84 (1.64-2.07)	<0.001	1.78 (1.58-2.01)	<0.001	1.02 (0.91-1.14)	0.742
Quartile 3 (14–19)	3.53 (3.02-4.11)	<0.001	3.32 (2.85-3.87)	<0.001	1.17 (1.00-1.36)	0.048
Quartile 4 (20–30)	9.78 (8.01-11.95)	<0.001	9.14 (7.47-11.17)	<0.001	1.74 (1.42-2.14)	<0.001
P-value for trend		<0.001		<0.001		<0.001
CNS symptoms score						
Quartile 1 (4)	0 [Reference]		0 [Reference]		0 [Reference]	
Quartile 2 (5–6)	1.49 (1.33-1.67)	<0.001	1.46 (1.31-1.64)	<0.001	1.03 (0.93-1.14)	0.616
Quartile 3 (7–9)	2.71 (2.34-3.15)	<0.001	2.61 (2.25-3.02)	<0.001	1.24 (1.09-1.41)	0.002
Quartile 4 (10–20)	6.3 (5.05-7.86)	<0.001	5.91 (4.75-7.35)	<0.001	1.61 (1.33-1.96)	<0.001
P-value for trend		<0.001		<0.001		<0.001

Model 1: Unadjusted for any potential covariates.

Model 2: Adjusted for age, sex, ethnicity, education, marital status, living arrangement, employment status, monthly household income, lifetime drinking status, lifetime smoking status, BMI, exercise habits, comorbidity.

Model 3: Additionally adjusted for PHQ-9 scores, GAD-7 scores, first episodes, antidepressant use, previous antidepressant use, sleep medications use, baseline SI, time trend.

BMI, body-mass index; PHQ-9, Patient Health Questionnaire-9; GAD-7, Generalized Anxiety Disorder-7; SI, suicidal ideation; OR, odds ratio; CI, confidence interval.

### 3.3 Restricted cubic spline analyses between somatic symptoms and SI

**Figure 2:**
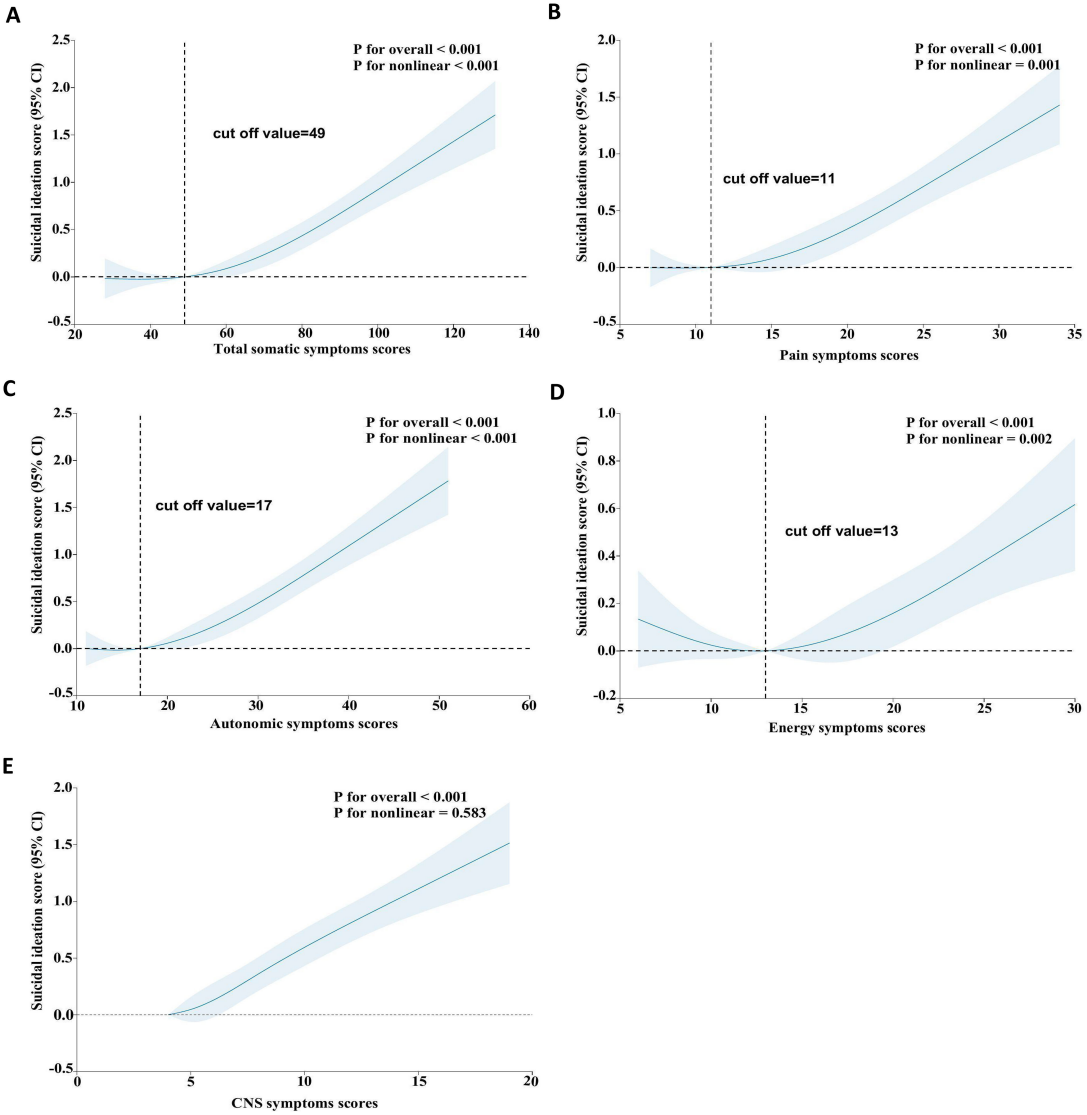
Restricted cubic spline analyses between somatic symptoms and SI. The model was adjusted for age, sex, ethnicity, education, marital status, living arrangement, employment status, monthly household income, lifetime drinking status, lifetime smoking status, weekly exercise habits, comorbidity, BMI, PHQ-9 scores, GAD-7 scores, first episodes, antidepressant use, previous antidepressant use, sleep medications use. BMI, body-mass index; PHQ-9, Patient Health Questionnaire-9; GAD-7, Generalized Anxiety Disorder-7; SI, suicidal ideation; OR, odds ratio; CI, confidence interval; CNS, central nervous system.

We found non-linear relationships between somatic symptom scores (e.g., total, pain, autonomic, and energy symptoms) and SI after adjusting for possible confounders (**Figures 2A–D**, all P-values for non-linearity < 0.05). Specifically, When the total somatic symptom score is below 49, the pain symptoms score is below 11, and the autonomic symptoms score is below 17, the risk of SI remains at a relatively stable and low level. However, when these scores exceeded the mentioned values, the risk of SI increases rapidly. Additionally, the risk of SI slightly decreases when the energy symptoms score is below 13, but it rises sharply once the score exceeds 13. In contrast, we observed linear associations between CNS symptoms scores and SI (**Figure 2E**, *P* for nonlinear = 0.583).

## 4 Discussion

In this longitudinal study, our findings suggest a significant association between somatic symptoms and their subtypes with SI among patients with MDD. Specifically, the somatic symptoms (e.g., total, painful, autonomic, and energy symptoms) significantly increased the risk of SI in a non-linear manner. In contrast, we observed linear associations between CNS symptoms and SI.

Our study found that total somatic symptoms were significantly associated with SI, independently of depressive symptom severity. These findings align with prior research (17, 23, 37–39). A meta-analysis of 33 studies demonstrated a significant association between somatic symptoms and increased risk of SI across diverse populations (37). Importantly, this increased risk was observed to be independent of any co-occurring mental disorders. Furthermore, a Japanese study focused on adolescents showed that increased somatic symptoms were significantly and independently associated with SI during mid-adolescence (38). This association remained significant after accounting for additional psychopathological and behavioral symptoms, as well as potential confounding factors. Another longitudinal study of 6,934 Chinese adolescents found that higher somatic symptom scores were more likely to report SI at baseline, with this tendency persisting at the 1-year follow-up (39). However, investigations focusing on patients with MDD remain confined to a handful of cross-sectional studies. For instance, a study of 217 Chinese patients with first-episode MDD found that those with more somatic symptoms had a greater risk of SI (17). Similarly, a Korean study of 811 outpatients with MDD found that patients with SI had significantly higher somatic symptom scores compared to those without SI (23). Although these studies varied in design, population, and assessment methods for somatic symptoms, SI, and risk factors, as well as in the number and type of somatic symptoms examined, their overall findings are consistent with ours. By using a longitudinal design and adjusting for multiple confounders—such as depression and anxiety severity, first-episode status, current and prior antidepressant use, sleep medications use, comorbid physical illness, and various health-related factors—our study provides more precise evidence on the association between somatic symptoms and SI in MDD patients.

Our study indicated that different subtypes of somatic symptoms (e.g., pain, autonomic, energy symptoms, and CNS symptoms) are significantly associated with SI. These results align with those of prior studies. For instance, a meta-analysis of 31 studies showed that individuals with any form of physical pain were more likely to report both current and lifetime SI, suicide plans, and attempts (9). A cross-sectional study conducted in Korea involving 414 patients with MDD found that patients with painful somatic symptoms had a 1.7 times higher risk of experiencing SI during the current depressive episode compared to those who did not report pain (40). Mao et al. studied MDD patients and found that specific pain symptoms (e.g., limb and pre-verbal pain) and certain autonomic symptoms (e.g., weight loss, increased appetite, hypersomnia, hyposexuality, and respiratory, circulatory, urinary, and sensory system symptoms) were significantly associated with SI (41). Jeon et al. found that MDD patients with SI exhibited significantly higher frequency and severity of somatic symptoms than those without SI, with current suicidal risk specifically associated with chest pain in men and neck or shoulder pain in women (23). Fang et al. found that among individuals with first-episode MDD, those with a higher number of somatic symptoms had an increased risk of SI, with pain symptoms, pre-verbal pain, and specific autonomic nervous system symptoms (e.g., late insomnia, weight loss, sensory system complaints, hypersomnia, and hyposexuality) associated with current SI (17). However, previous cross-sectional studies of MDD patients have mainly examined associations between single symptoms within specific somatic subtypes and SI, suggesting that a single-symptom perspective may underestimate the overall risk. Given that individuals with MDD frequently present with multiple concurrent somatic symptoms, the overall burden within each subtype requires systematic evaluation. This study offers a more comprehensive and accurate evidence by calculating total scores for four somatic subtypes to assess their overall burden and relationship with SI. This enables more effective identification of high - risk individuals and the development of more targeted management strategies.

In addition, we found a significant nonlinear relationship between total somatic symptoms and SI. When somatic symptoms are mild, the risk of SI remains low and stable. However, as symptoms worsen, the risk of SI increases significantly. This nonlinear relationship can help establish a treatment threshold. specifically, a score of 49 could serve as a practical cutoff for initiating somatic-targeted interventions in clinical practice. Similar nonlinear patterns were observed for pain, autonomic, and energy symptom subtypes. Defining distinct thresholds for each subtype enables more precise risk assessment. Notably, no nonlinear dose-response relationship was observed between CNS symptoms and SI. This may be related to the multidimensionality and complexity of central nervous system symptoms. The underlying mechanisms warrant further in-depth investigation.

Individuals with MDD who have somatic symptoms exhibit more complex clinical manifestations than those without somatic symptoms (42). These individuals usually have a more severe condition, a longer disease course, and a lower quality of life (16, 43). Persistent severe somatic symptoms not only indicate clinically significant impairment (44), but also significantly affect daily activities and functional levels (45). Furthermore, the severity of somatic symptoms may trigger health anxiety and concerns, which in turn may further increase the risk of SI (46).

The mechanistic association between somatic symptoms and SI in MDD is underpinned by multi-system dysregulation. Peripheral and central elevations of pro-inflammatory cytokines (TNF-α, IL-6) (47–52), hyperactivity of the HPA axis (47, 53), dysfunctions in the serotonergic and noradrenergic neurotransmitter systems (54–56), alterations in cerebral structure—such as regional grey-matter volume reductions and aberrant functional activity (47, 53, 57–59)—interact synergistically. These interactions form a complex pathophysiological network that influences the link between somatic symptoms and SI in patients with MDD.

Our results underscore the necessity of tackling somatic symptoms in MDD management. Early identification of clinical features that increase disease burden and management complexity is crucial for preventing depression from progressing to treatment-resistant and suicidal trajectories, as highlighted by Fiorillo et al. (60). Establishing subtype-specific thresholds allows for more precise risk stratification. In line with the international consensus by Maina et al. (61), patients with a high somatic burden should undergo multidimensional assessment and proactive suicide-risk monitoring. We recommend a total somatic score ≥49 as a practical threshold to trigger intensified surveillance and early initiation of symptom evaluation and targeted interventions, so as to prevent the progression of depression toward treatment resistance and suicidal trajectories. Clinicians should routinely and comprehensively assess somatic symptoms, incorporate symptom-targeted strategies into treatment plans, select antidepressants with analgesic benefits or minimal physical symptom exacerbation based on individual patient profiles (62), and combine these approaches with evidence-based non-pharmacological interventions, such as cognitive-behavioral therapy, exercise therapy, and other pharmacological treatments (63–65). Timely identification and effective management of somatic symptoms and their subtypes are essential for improving depression prognosis and reducing suicidal behavior.

Our study has several notable strengths. Firstly, to our knowledge, this is the first longitudinal study to explore the relationship between somatic symptoms and SI in patients with MDD. Secondly, our study examines the relationship between somatic symptoms and SI across four major subtypes: pain symptoms, autonomic nervous system symptoms, energy symptoms, and CNS symptoms. This is in contrast to previous studies that often focused on specific symptoms within subtypes. Thirdly, we identified potential inflection points in the non-linear relationships between somatic symptoms and SI. This is crucial for understanding the complex dynamics underlying these associations and informing targeted interventions.

When interpreting and generalizing our findings, several limitations must be considered. Firstly, the study was based in a hospital setting, which might restrict the results to be generalized to patients who do not seek treatment in hospitals. Secondly, we used the SSI scale to assess the 28 types of somatic symptoms in patients and did not measure or analyze other somatic symptoms that might be present in our study population. Thirdly, since we relied on self-report measures to assess somatic symptoms, SI, and depressive symptoms, recall bias could not be entirely avoided. Fourthly, although a longitudinal design was employed, the possibility of unmeasured confounders and reverse causation limits causal inferences.

## 5 Conclusion

Our study shows that somatic symptoms and their subtypes are significantly associated with SI in MDD patients. A critical threshold at ≥ 49 points on the total somatic symptom score indicates significantly elevated suicide risk. We recommend: (1) routine standardized assessment of somatic symptoms in all MDD patients;(2) implementation of targeted interventions for patients exceeding this threshold; and (3) enhanced suicide risk monitoring for these patients. This evidence-based stratified management approach holds significant clinical value for improving depression outcomes and suicide prevention.

## Data Availability

The raw data supporting the conclusions of this article will be made available by the authors, without undue reservation.

## References

[1] Marx W Penninx B Solmi M Furukawa TA Firth J Carvalho AF. Major depressive disorder. Nat Rev Dis Primers. (2023) 9:44. doi: 10.1038/s41572-023-00454-1, PMID: 37620370

[2] Maj M Stein DJ Parker G Zimmerman M Fava GA De Hert M. The clinical characterization of the adult patient with depression aimed at personalization of management. World psychiatry: Off J World Psychiatr Assoc (WPA). (2020) 19:269–93. doi: 10.1002/wps.20771, PMID: 32931110 PMC7491646

[3] Collaborators C-MD. Global prevalence and burden of depressive and anxiety disorders in 204 countries and territories in 2020 due to the COVID-19 pandemic. Lancet (London England). (2021) 398:1700–12. doi: 10.1016/s0140-6736(21)02143-7, PMID: 34634250 PMC8500697

[4] GBD 2021 Diseases and Injuries Collaborators. Global incidence, prevalence, years lived with disability (YLDs), disability-adjusted life-years (DALYs), and healthy life expectancy (HALE) for 371 diseases and injuries in 204 countries and territories and 811 subnational locations, 1990-2021: a systematic analysis for the Global Burden of Disease Study 2021. Lancet (London England). (2024) 403(10440):2133–61. doi: 10.1016/s0140-6736(24)00757-8, PMID: 38642570 PMC11122111

[5] Zou S Song X Tan W Deng F Zhang H Xu H. Xiong P et al: Core self-evaluation as mediator between depressive symptoms and suicidal ideation in adolescents. J Affect Disord. (2022) 302:361–6. doi: 10.1016/j.jad.2022.01.093, PMID: 35104465

[6] Friedrich MJ. Depression is the leading cause of disability around the world. Jama. (2017) 317:1517. doi: 10.1001/jama.2017.3826, PMID: 28418490

[7] World Health Organization. (2024). Suicide. Available online at: https://www.who.int/zh/news-room/fact-sheets/detail/suicide (Accessed May 23, 2025).

[8] Cai H Jin Y Liu S Zhang Q Zhang L Cheung T. Prevalence of suicidal ideation and planning in patients with major depressive disorder: A meta-analysis of observation studies. J Affect Disord. (2021) 293:148–58. doi: 10.1016/j.jad.2021.05.115, PMID: 34192629

[9] Calati R Laglaoui Bakhiyi C Artero S Ilgen M Courtet P. The impact of physical pain on suicidal thoughts and behaviors: Meta-analyses. J Psychiatr Res. (2015) 71:16–32. doi: 10.1016/j.jpsychires.2015.09.004, PMID: 26522868

[10] Dong M Wang SB Li Y Xu DD Ungvari GS Ng CH. Prevalence of suicidal behaviors in patients with major depressive disorder in China: A comprehensive meta-analysis. J Affect Disord. (2018) 225:32–9. doi: 10.1016/j.jad.2017.07.043, PMID: 28779680

[11] Hu S Mo D Guo P Zheng H Jiang X Zhong H. Correlation between suicidal ideation and emotional memory in adolescents with depressive disorder. Sci Rep. (2022) 12:5470. doi: 10.1038/s41598-022-09459-4, PMID: 35361837 PMC8971389

[12] Vaccarino AL Sills TL Evans KR Kalali AH. Prevalence and association of somatic symptoms in patients with Major Depressive Disorder. J Affect Disord. (2008) 110:270–6. doi: 10.1016/j.jad.2008.01.009, PMID: 18280580

[13] Bekhuis E Boschloo L Rosmalen JG Schoevers RA. Differential associations of specific depressive and anxiety disorders with somatic symptoms. J psychosomatic Res. (2015) 78:116–22. doi: 10.1016/j.jpsychores.2014.11.007, PMID: 25524436

[14] Ryder AG Yang J Zhu X Yao S Yi J Heine SJ. The cultural shaping of depression: somatic symptoms in China, psychological symptoms in North America? J Abnormal Psychol. (2008) 117:300–13. doi: 10.1037/0021-843x.117.2.300, PMID: 18489206

[15] Parker G Cheah YC Roy K. Do the Chinese somatize depression? A cross-cultural study. Soc Psychiatry Psychiatr Epidemiol. (2001) 36:287–93. doi: 10.1007/s001270170046, PMID: 11583458

[16] Novick D Montgomery W Aguado J Kadziola Z Peng X Brugnoli R. Which somatic symptoms are associated with an unfavorable course in Asian patients with major depressive disorder? J Affect Disord. (2013) 149:182–8. doi: 10.1016/j.jad.2013.01.020, PMID: 23521872

[17] Fang X Zhang C Wu Z Peng D Xia W Xu J. The association between somatic symptoms and suicidal ideation in Chinese first-episode major depressive disorder. J Affect Disord. (2019) 245:17–21. doi: 10.1016/j.jad.2018.10.110, PMID: 30366233

[18] Rasmussen S Jensen CT Rosendal M Vægter HB Søndergaard J Jarbøl DE. Multiple physical symptoms and individual characteristics - A cross-sectional study of the general population. J psychosomatic Res. (2020) 131:109941. doi: 10.1016/j.jpsychores.2020.109941, PMID: 32050120

[19] Joustra ML Janssens KA Bültmann U Rosmalen JG. Functional limitations in functional somatic syndromes and well-defined medical diseases. Results from the general population cohort LifeLines. J psychosomatic Res. (2015) 79:94–9. doi: 10.1016/j.jpsychores.2015.05.004, PMID: 26026696

[20] Jiang Y Zhu D Huang X Li Y Chen Y Jiang Y. Liao Y et al: Associations between somatic symptoms and remission of major depressive disorder: A longitudinal study in China. J Psychiatr Res. (2024) 172:382–90. doi: 10.1016/j.jpsychires.2024.02.056, PMID: 38452636

[21] McIntyre RS Konarski JZ Mancini DA Zurowski M Giacobbe P Soczynska JK. Improving outcomes in depression: a focus on somatic symptoms. J psychosomatic Res. (2006) 60:279–82. doi: 10.1016/j.jpsychores.2005.09.010, PMID: 16516660

[22] Su Y Ye C Xin Q Si T. Major depressive disorder with suicidal ideation or behavior in Chinese population: A scoping review of current evidence on disease assessment, burden, treatment and risk factors. J Affect Disord. (2023) 340:732–42. doi: 10.1016/j.jad.2023.08.106, PMID: 37619652

[23] Jeon HJ Woo JM Kim HJ Fava M Mischoulon D Cho SJ. Yoo I et al: Gender Differences in Somatic Symptoms and Current Suicidal Risk in Outpatients with Major Depressive Disorder. Psychiatry Invest. (2016) 13:609–15. doi: 10.4306/pi.2016.13.6.609, PMID: 27909451 PMC5128348

[24] Oztürk E Sar V. Somatization as a predictor of suicidal ideation in dissociative disorders. Psychiatry Clin Neurosci. (2008) 62:662–8. doi: 10.1111/j.1440-1819.2008.01865.x, PMID: 19068002

[25] Novick D Montgomery WS Aguado J Peng X Brugnoli R Haro JM. Which somatic symptoms are associated with an unfavorable course in Chinese patients with major depressive disorder? Asia-Pacific psychiatry: Off J Pacific Rim Coll Psychiatrists. (2015) 7:427–35. doi: 10.1111/appy.12189, PMID: 26047023

[26] Zhang H Liao Y Han X Fan B Liu Y Lui LMW. Guo L et al: Screening Depressive Symptoms and Incident Major Depressive Disorder Among Chinese Community Residents Using a Mobile App-Based Integrated Mental Health Care Model: Cohort Study. J Med Internet Res. (2022) 24:e30907. doi: 10.2196/30907, PMID: 35594137 PMC9166637

[27] Wu X Zhu Y Wu Z Huang J Cao L Wang Y. Yao Z et al: Identifying the Subtypes of Major Depressive Disorder Based on Somatic Symptoms: A Longitudinal Study Using Latent Profile Analysis. Front Psychiatry. (2022) 13:759334. doi: 10.3389/fpsyt.2022.759334, PMID: 35903631 PMC9314656

[28] Kroenke K Spitzer RL Williams JB Linzer M Hahn SR deGruy FV 3rd. Brody D: Physical symptoms in primary care. Predictors of psychiatric disorders and functional impairment. Arch Family Med. (1994) 3:774–9. doi: 10.1001/archfami.3.9.774, PMID: 7987511

[29] Li X Zhang H Han X Guo L Ceban F Liao Y. Song W et al: Predictive potential of somatic symptoms for the identification of subthreshold depression and major depressive disorder in primary care settings. Front Psychiatry. (2023) 14:999047. doi: 10.3389/fpsyt.2023.999047, PMID: 36865073 PMC9971499

[30] Beck AT Kovacs M Weissman A. Assessment of suicidal intention: the Scale for Suicide Ideation. J consulting Clin Psychol. (1979) 47:343–52. doi: 10.1037//0022-006x.47.2.343, PMID: 469082

[31] Zhang J Brown GK. Psychometric properties of the scale for suicide ideation in China. Arch suicide research: Off J Int Acad Suicide Res. (2007) 11:203–10. doi: 10.1080/13811110600894652, PMID: 17453698 PMC3210860

[32] Chen Y Han X Jiang Y Jiang Y Huang X Wang W. Longitudinal association between stressful life events and suicidal ideation in adults with major depression disorder: the mediating effects of insomnia symptoms. Behav Sci (Basel Switzerland). (2024) 14(6):467. doi: 10.3390/bs14060467, PMID: 38920799 PMC11200868

[33] Huang X Fan B Jiang Y Li Y Chen Y Zhao H. Chen Y et al: Associations of rumination with suicidal ideation and suicide attempts amongst individuals with major depressive disorder: A 12-month longitudinal study in China. Compr Psychiatry. (2024) 132:152472. doi: 10.1016/j.comppsych.2024.152472, PMID: 38513451

[34] Liao YH Fan BF Zhang HM Guo L Lee Y Wang WX. The impact of COVID-19 on subthreshold depressive symptoms: a longitudinal study. Epidemiol Psychiatr Sci. (2021) 30:e20. doi: 10.1017/s2045796021000044, PMID: 33583474 PMC7985630

[35] Li Y Chen Y Jiang Y Wang W Guo L Fan B. Teopiz KM et al: Associations of childhood trauma with remission and treatment response after 12 weeks of selective serotonin reuptake inhibitor treatment in patients with major depressive disorder. Gen Hosp Psychiatry. (2025) 92:12–9. doi: 10.1016/j.genhosppsych.2024.12.002, PMID: 39662212

[36] Shi J Han X Liao Y Zhao H Fan B Zhang H. Guo L et al: Associations of stressful life events with subthreshold depressive symptoms and major depressive disorder: The moderating role of gender. J Affect Disord. (2023) 325:588–95. doi: 10.1016/j.jad.2023.01.050, PMID: 36657495

[37] Torres ME Löwe B Schmitz S Pienta JN van der Feltz-Cornelis C Fiedorowicz JG. Suicide and suicidality in somatic symptom and related disorders: A systematic review. J psychosomatic Res. (2021) 140:110290. doi: 10.1016/j.jpsychores.2020.110290, PMID: 33227556 PMC7945369

[38] Uno A Nagaoka D Usami S Yamaguchi S Minami R Tanaka R. Miyashita M et al: Suicidal Thoughts and Trajectories of Psychopathological and Behavioral Symptoms in Adolescence. JAMA network Open. (2024) 7:e2353166. doi: 10.1001/jamanetworkopen.2023.53166, PMID: 38270951 PMC10811562

[39] Ji Y Liu ZZ Jia CX Liu X. Associations between somatic symptoms and suicidal behavior: a cohort study in Chinese adolescents. BMC Psychol. (2025) 13:788. doi: 10.1186/s40359-025-03113-0, PMID: 40665454 PMC12261720

[40] Bahk WM Park S Jon DI Yoon BH Min KJ Hong JP. Relationship between painful physical symptoms and severity of depressive symptomatology and suicidality. Psychiatry Res. (2011) 189:357–61. doi: 10.1016/j.psychres.2011.01.009, PMID: 21329990

[41] Mao R Xu J Peng D Chen J Wu Z Fang Y. The role of gender factors influencing multiple dimensions of somatic symptoms in major depressive disorder patients with suicidal ideation: insights from the Chinese NSSD study. BMC Psychiatry. (2024) 24:732. doi: 10.1186/s12888-024-06172-6, PMID: 39456015 PMC11515138

[42] Liu JJ Huang X Bao YP Lu L Dong P Wolkowitz OM. Painful physical symptoms and antidepressant treatment outcome in depression: a systematic review and meta-analysis. Mol Psychiatry. (2024) 29:2560–7. doi: 10.1038/s41380-024-02496-7, PMID: 38480874

[43] Bekhuis E Boschloo L Rosmalen JG de Boer MK Schoevers RA. The impact of somatic symptoms on the course of major depressive disorder. J Affect Disord. (2016) 205:112–8. doi: 10.1016/j.jad.2016.06.030, PMID: 27428090

[44] Creed FH Davies I Jackson J Littlewood A Chew-Graham C Tomenson B. The epidemiology of multiple somatic symptoms. J psychosomatic Res. (2012) 72:311–7. doi: 10.1016/j.jpsychores.2012.01.009, PMID: 22405227

[45] Toussaint A Kroenke K Baye F Lourens S. Comparing the Patient Health Questionnaire - 15 and the Somatic Symptom Scale - 8 as measures of somatic symptom burden. J psychosomatic Res. (2017) 101:44–50. doi: 10.1016/j.jpsychores.2017.08.002, PMID: 28867423

[46] Tomenson B Essau C Jacobi F Ladwig KH Leiknes KA Lieb R. Rief W et al: Total somatic symptom score as a predictor of health outcome in somatic symptom disorders. Br J psychiatry: J Ment Sci. (2013) 203:373–80. doi: 10.1192/bjp.bp.112.114405, PMID: 24072756

[47] Rief W Hennings A Riemer S Euteneuer F. Psychobiological differences between depression and somatization. J psychosomatic Res. (2010) 68:495–502. doi: 10.1016/j.jpsychores.2010.02.001, PMID: 20403510

[48] Rosenblat JD Cha DS Mansur RB McIntyre RS. Inflamed moods: a review of the interactions between inflammation and mood disorders. Prog Neuropsychopharmacol Biol Psychiatry. (2014) 53:23–34. doi: 10.1016/j.pnpbp.2014.01.013, PMID: 24468642

[49] Milaneschi Y Kappelmann N Ye Z Lamers F Moser S Jones PB. Association of inflammation with depression and anxiety: evidence for symptom-specificity and potential causality from UK Biobank and NESDA cohorts. Mol Psychiatry. (2021) 26:7393–402. doi: 10.1038/s41380-021-01188-w, PMID: 34135474 PMC8873022

[50] Zunszain PA Hepgul N Pariante CM. Inflammation and depression. Curr topics Behav Neurosci. (2013) 14:135–51. doi: 10.1007/7854_2012_211, PMID: 22553073

[51] Sha Q Fu Z Escobar Galvis ML Madaj Z Underwood MD Steiner JA. Rozoklija G et al: Integrative transcriptome- and DNA methylation analysis of brain tissue from the temporal pole in suicide decedents and their controls. Mol Psychiatry. (2024) 29:134–45. doi: 10.1038/s41380-023-02311-9, PMID: 37938766 PMC11078738

[52] Black C Miller BJ. Meta-analysis of cytokines and chemokines in suicidality: distinguishing suicidal versus nonsuicidal patients. Biol Psychiatry. (2015) 78:28–37. doi: 10.1016/j.biopsych.2014.10.014, PMID: 25541493

[53] Deininger-Czermak E Spencer L Zoelch N Sankar A Gascho D Guggenberger R. Magnetic resonance imaging of regional gray matter volume in persons who died by suicide. Mol Psychiatry. (2025) 30:1029–33. doi: 10.1038/s41380-024-02730-2, PMID: 39237718 PMC11835744

[54] Liu Y Zhao J Fan X Guo W. Dysfunction in serotonergic and noradrenergic systems and somatic symptoms in psychiatric disorders. Front Psychiatry. (2019) 10:286. doi: 10.3389/fpsyt.2019.00286, PMID: 31178761 PMC6537908

[55] Stahl S Briley M. Understanding pain in depression. Hum Psychopharmacol. (2004) 1:S9–S13. doi: 10.1002/hup.619, PMID: 15378669

[56] Mann JJ Currier D Stanley B Oquendo MA Amsel LV Ellis SP. Can biological tests assist prediction of suicide in mood disorders? Int J Neuropsychopharmacol. (2006) 9:465–74. doi: 10.1017/s1461145705005687, PMID: 15967058

[57] Mo D Guo P Hu S Tao R Zhong H Liu H. Characteristics and correlation of gray matter volume and somatic symptoms in adolescent patients with depressive disorder. Front Psychiatry. (2023) 14:1197854. doi: 10.3389/fpsyt.2023.1197854, PMID: 37559918 PMC10407247

[58] Jacob L Haro JM Koyanagi A. The association between pain and suicidal behavior in an English national sample: The role of psychopathology. J Psychiatr Res. (2018) 98:39–46. doi: 10.1016/j.jpsychires.2017.12.007, PMID: 29274531

[59] de Kloet ER Derijk RH Meijer OC. Therapy Insight: is there an imbalanced response of mineralocorticoid and glucocorticoid receptors in depression? Nat Clin Pract Endocrinol Metab. (2007) 3:168–79. doi: 10.1038/ncpendmet0403, PMID: 17237843

[60] Fiorillo A Demyttenaere K Martiadis V Martinotti G. Editorial: Treatment resistant depression (TRD): epidemiology, clinic, burden and treatment. Front Psychiatry. (2025) 16:1588902. doi: 10.3389/fpsyt.2025.1588902, PMID: 40171309 PMC11958960

[61] Maina G Adami M Ascione G Bondi E De Berardis D Delmonte D. Ottavianelli E et al: Nationwide consensus on the clinical management of treatment-resistant depression in Italy: a Delphi panel. Ann Gen Psychiatry. (2023) 22:48. doi: 10.1186/s12991-023-00478-7, PMID: 37996836 PMC10668442

[62] IsHak WW Wen RY Naghdechi L Vanle B Dang J Knosp M. Eskander L et al: Pain and Depression: A Systematic Review. Harvard Rev Psychiatry. (2018) 26:352–63. doi: 10.1097/hrp.0000000000000198, PMID: 30407234

[63] Haun MW van Eickels D Tönnies J Graue L Ayoub-Schreifeldt M Wensing M. An integrated mental health video consultations model for patients with somatic symptom disorder in primary care: The randomized VISION pilot trial. J psychosomatic Res. (2024) 182:111801. doi: 10.1016/j.jpsychores.2024.111801, PMID: 38761536

[64] Löwe B Toussaint A Rosmalen JGM Huang WL Burton C Weigel A. Persistent physical symptoms: definition, genesis, and management. Lancet (London England). (2024) 403:2649–62. doi: 10.1016/s0140-6736(24)00623-8, PMID: 38879263

[65] Danielsson L Papoulias I Petersson EL Carlsson J Waern M. Exercise or basic body awareness therapy as add-on treatment for major depression: a controlled study. J Affect Disord. (2014) 168:98–106. doi: 10.1016/j.jad.2014.06.049, PMID: 25043321

